# The combined effect of chemoprophylaxis with single dose rifampicin and immunoprophylaxis with BCG to prevent leprosy in contacts of newly diagnosed leprosy cases: a cluster randomized controlled trial (MALTALEP study)

**DOI:** 10.1186/1471-2334-13-456

**Published:** 2013-10-03

**Authors:** Renate A Richardus, Khorshed Alam, David Pahan, Sabiena G Feenstra, Annemieke Geluk, Jan H Richardus

**Affiliations:** 1 Department of Public Health, Erasmus MC, University Medical Center Rotterdam, P.O. Box 2040, 3000, CA Rotterdam, The Netherlands; 2 Rural Health Program, The Leprosy Mission International Bangladesh, Nilphamari, Bangladesh; 3 Department of Infectious Diseases, Leiden University Medical Center, Leiden, The Netherlands

**Keywords:** Leprosy, *M. leprae*, BCG vaccine, Rifampicin, Prevention, RCT, Study protocol

## Abstract

**Background:**

Despite almost 30 years of effective chemotherapy with MDT, the global new case detection rate of leprosy has remained quite constant over the past years. New tools and methodologies are necessary to interrupt the transmission of *M. leprae*. Single-dose rifampicin (SDR) has been shown to prevent 57% of incident cases of leprosy in the first two years, when given to contacts of newly diagnosed cases. Immunization of contacts with BCG has been less well documented, but appears to have a preventive effect lasting up to 9 years. However, one major disadvantage is the occurrence of excess cases within the first year after immunization. The objective of this study is to examine the effect of chemoprophylaxis with SDR and immunoprophylaxis with BCG on the clinical outcome as well as on host immune responses and gene expression profiles in contacts of newly diagnosed leprosy patients. We hypothesize that the effects of both interventions may be complementary, causing the combined preventive outcome to be significant and long-lasting.

**Methods/design:**

Through a cluster randomized controlled trial we compare immunization with BCG alone with BCG plus SDR in contacts of new leprosy cases. Contact groups of around 15 persons will be established for each of the 1300 leprosy patients included in the trial, resulting in approximately 20,000 contacts in total. BCG will be administered to the intervention group followed by SDR, 2 months later. The control group will receive BCG only. In total 10,000 contacts will be included in both intervention arms over a 2-year period. Follow-up will take place one year as well as two years after intake. The primary outcome is the occurrence of clinical leprosy within two years. Simultaneously with vaccination and SDR, blood samples for *in vitro* analyses will be obtained from 300 contacts participating in the trial to determine the effect of these chemo- and immunoprophylactic interventions on immune and genetic host parameters.

**Discussion:**

Combined chemoprophylaxis and immunoprophylaxis is potentially a very powerful and innovative tool aimed at contacts of leprosy patients that could reduce the transmission of *M. leprae* markedly. The trial intends to substantiate this potential preventive effect. Evaluation of immune and genetic biomarker profiles will allow identification of pathogenic versus (BCG-induced) protective host biomarkers and could lead to effective prophylactic interventions for leprosy using optimized tools for identification of individuals who are most at risk of developing disease.

**Trial registration:**

Netherlands Trial Register: NTR3087

## Background

The global number of new leprosy cases has remained constant over the past years [1], indicating that transmission of *Mycobacterium leprae,* the causative agent of leprosy, is ongoing in many endemic countries. The basic intervention is multidrug therapy (MDT) given to newly found leprosy cases, but this seems to be insufficient to decrease the number of new cases.

The main risk of exposure to *M. leprae* is in close contacts of new, untreated cases. Epidemiological studies have shown that the chance of finding a previously undiagnosed leprosy patient is ten times higher in household contacts of leprosy patients than in the general population, and the chance of finding leprosy among different categories of neighbors and social contacts is between three and five-fold [2,3]. Therefore, it has been suggested that contacts should be the main focus of a future leprosy control strategy. Such strategy should have three basic pillars: 1) case detection; 2) case management; and 3) contact management [4].

In the past years, many studies have investigated the use of immunoprophylaxis (vaccination) and chemoprophylaxis to prevent leprosy. These interventions have focused primarily on contacts of leprosy patients. Bacillus Calmette-Guérin (BCG) vaccination is known as a vaccine against tuberculosis and is routinely given to infants as part of the neonatal immunization scheme in many parts of the world. BCG is also recognized as protecting against leprosy [5,6]. Over the years several vaccine trials using BCG have been performed to establish its protective effect against leprosy, often in combination with *M. leprae* or related mycobacterium vaccines. BCG was as good as, or superior to the other mycobacterium vaccines [4].

BCG efficacy appeared to be significantly higher in studies when BCG vaccination was targeting household contacts of leprosy patients compared with the ones conducted in the general population: 68% *vs.* 53% [5]. In Brazil, the government officially recommends BCG (re)vaccination to protect household contacts of leprosy cases. This policy was assessed in a cohort study showing that the protection conferred by BCG was 56% and was not substantially affected by previous BCG vaccination [7]. The risk of tuberculoid leprosy during the initial months was high among those vaccinated with no previous BCG vaccination; 21 of 58 new leprosy cases (36%) occurred in the first year. This risk, however, had substantially declined by the first year and in the following years the protection rate in this group reached 80% [7]. The results of this study are not conclusive due to some methodological inconsistencies. In particular, the issue of increased risk of tuberculoid leprosy in the first months after BCG vaccination needs further evaluation.

With regard to chemoprophylaxis, the COLEP study in Bangladesh showed that the use of a single dose of rifampicin (SDR) in contacts of newly diagnosed leprosy patients reduced the overall incidence of leprosy in the first two years with 57% [8]. Furthermore, this study showed that the effect of SDR depended on the BCG status of the contact [9]: If the contact had received BCG vaccination as part of a childhood vaccination program (as established by the presence of a BCG-scar), the protective effect of SDR was 80%. Childhood BCG vaccination and SDR each have a protective effect in contacts of approximately 60%, but if contacts who had previously received BCG vaccination also received SDR, the protective effect appeared to be additive.

Based on the experiences with BCG vaccination and SDR chemoprophylaxis in preventing leprosy among contacts of leprosy patients, a trial was initiated in Bangladesh to assess the efficacy of a combined strategy (acronym: MALTALEP study, named after the main sponsor of the research project). The objective of this paper is to describe the design of a cluster randomized controlled trial, in which contacts of newly diagnosed leprosy patients will either receive BCG alone, or BCG plus SDR. In particular, it is important to determine whether the excess cases in the first year after immunoprophylaxis can be prevented by chemoprophylaxis, while maintaining the protective effect.

## Methods/design

### Objectives and hypothesis

The objective of this study is to examine the combined effect of chemoprophylaxis with single dose rifampicin (SDR) and immunoprophylaxis with Bacillus Calmette-Guérin (BCG), in contacts of new cases of leprosy. Both interventions are known to have a preventive effect and we hypothesize that these effects may be complementary, so that the combined effect may be significant and long-lasting.

### Study design

The intervention consists of a cluster randomized controlled trial, with two treatment arms, to study the effectiveness of the BCG vaccine versus BCG in combination with SDR in the prevention of leprosy among contacts of newly diagnosed leprosy patients.

### Setting

The study takes place in the districts of Nilphamari, Rangpur, Thakurgaon and Panchagarh in Northwest Bangladesh. Patients will enter into the trial through the Rural Health Program (RHP) of The Leprosy Mission International Bangladesh (TLMIB), located at the Nilphamari Hospital; a referral hospital specialized in the detection and treatment of leprosy. The population of the four districts is around 7,000,000 (2011 census [10]) and 800–900 new leprosy patients are detected per year. The population in the four districts is mainly rural, but also includes six main towns.

### Participants

Newly diagnosed leprosy patients will be included in the trial after being diagnosed with leprosy according to the Rural Health Program guidelines, which follow those of the National Leprosy Control Program [11,12]. All new leprosy patients are confirmed by a medical officer, and this confirmation is written on the patient card. Around 1,300 consecutive leprosy patients will be enrolled into the study. After a patient is diagnosed, patient details will be recorded (Table 1). Multidrug therapy (MDT) will be started according to the national guidelines. Intake of single-lesion PB (SLPB) patients will be stopped when 500 such patients have been included; the same will apply to the group of other PB patients (PB2-5, with two to five skin lesions on physical examination). This will ensure an intake of at least 300 multibacillary (MB) patients. Within two weeks after the new leprosy patient has received the second dose of MDT (four weeks after the first dose), a survey will be performed among all household contacts. During this survey, contact groups will be formed consisting of approximately 15 persons for each patient. Thus, the total number of contacts included will be around 20,000.

**Table 1:** Patient and contact data recorded

1	Personal data of patient and all selected contacts: name, year of birth, sex and relation of contact to the selected patient
2	Brief information regarding medical history of all contacts (liver disease, malignancies, HIV, TB, leprosy, pregnancy, vaccination status and medication use) to ensure that the participants have no contraindications for BCG vaccination or use of the medicine rifampicin
3	Results of physical examination on signs and symptoms of leprosy (including leprosy classification and WHO disability grade) and actions taken accordingly
4	Interventions: BCG vaccination, medication provided, blood sample taken
5	Record of any adverse reactions and actions taken accordingly
6	Report of follow up visits

Exclusion criteria for patients are as follows: any patient who refuses examination of contacts, any patient who suffers from the pure neural form of leprosy, any patient who resides only temporarily in the study area, any new patient found during contact examination of the index case, any new patient living less than 100 m away from a patient already included in the study or first and second degree relatives of a patient already included in the study.

The following categories of contacts of new leprosy patients have been distinguished for inclusion: those living in the same house (household members), those living in a house on the same compound, sharing the same kitchen, and direct neighbors (first neighbors). Exclusion criteria for contacts are as follows: any person who refuses informed consent, any woman indicating that she is pregnant, any person currently on TB or leprosy treatment, any person below 5 years of age, any person known to suffer from liver disease or jaundice, any person residing temporarily in the area, any person suffering from leprosy at the initial survey (these patients will be referred to the clinic for leprosy treatment) and any person who is a contact of another patient and is already enrolled in the contact group of the other patient.

### Randomization

Each contact group will be randomly allocated to one of the two study arms (Arm 1: BCG only, or Arm 2: BCG plus SDR) by means of computer generation with a 1:1 ratio for each arm. The allocation to receive SDR is stamped on the data collection forms of each contact group. Immunoprophylaxis with BCG will be given at the moment of the contact survey to all included contacts in both arms of the trial, followed by chemoprophylaxis with SDR eight weeks later in contacts of Arm 2.

**Figure 1:**
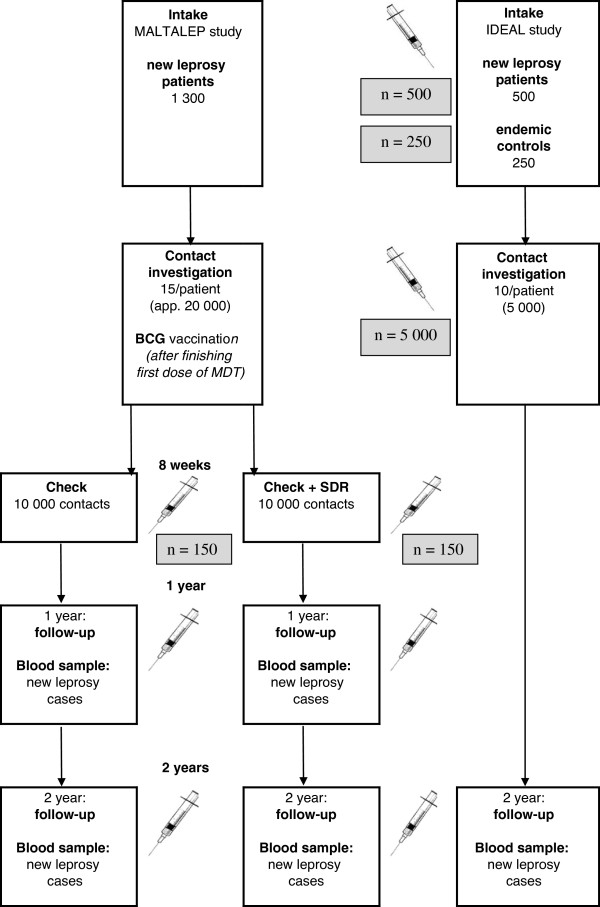
Schematic representation of the trial (MALTALEP study), together with the blood samples taken for analysis of host immune and gene profiles from subjects in the trial and in a non-intervention group (IDEAL study).

A schematic representation of the MALTALEP study is given in Figure 1 (left side), together with a non-intervention group (right side) and the sampling framework for analysis of host immune and gene profiles, which is part of the IDEAL study (see below).

### Outcome measures

The primary outcome measure is the number of new leprosy patients emerging from the contact groups. The proportions between the two arms of the trial will be compared after one and two years.

Secondary data analysis will be carried out in order to define special groups at risk for developing leprosy and blood sample analysis of host immune and gene profiles.

### Intervention implementation and data collection

The medication provided in the trial is rifampicin. Rifampicin comes in capsules of 150 mg and the dosage is the same as recommended in the guidelines of the national leprosy control program of Bangladesh and RHP (Table 2). According to body weight and age, 2 to 4 capsules are taken by the contact under direct supervision of a RHP staff member.

**Table 2:** Dosage of rifampicin chemoprophylaxis according to age and body weight

**Age/weight**	**Dose of rifampicin**
Adult >35 kg	600 mg
Adult <35 kg	450 mg
Child 10–14 years	450 mg
Child 5–9 years	300 mg

The vaccine provided in the trial is BCG. The BCG vaccine is applied by trained research assistants to all included contacts. 0.1 ml of BCG vaccine is given by intradermal injection. The BCG vaccine used in the trial (and in routine neonatal vaccination in Bangladesh) is produced at the Japan BCG Laboratory and is a freeze-dried glutamate BCG vaccine (Japan), composed of 0,5 mg/ampule live bacteria of Calmette-Guérin (as approximately 70% moist bacteria) and 2,0 mg/ampule sodium glutamate (as a stabilizer). Vaccines are stored at the State Immunisation Programme facilities.

All eligible patients and their contacts will be informed verbally about the study through the reading of the consent form, and then invited to participate. Before inclusion, the patient and their contacts are asked to sign a form if they agree to participate in the study. For illiterate people a thumb print will be taken, and for minors under 16 years of age, the guardian’s additional consent will be taken. Contacts explicitly give consent for BCG vaccination and SDR, and for blood drawing. Furthermore, the researcher has to sign that he/she has accurately read or witnessed the accurate reading of the consent form to the participants, that the individuals have had the opportunity to ask questions and they have given consent freely. Participants will also be informed that they will be offered free consultation and treatment in the case of adverse events following BCG vaccination. They are provided with a vaccination card with details on how to reach the researcher if they have any concerns. Also, participants are informed that their participation is completely voluntary and that they may choose not to participate or stop at any point of time. Their decision not to volunteer, or to refuse to answer particular questions, will not affect their relationship with the researchers or other staff members of RHP.

At the initial contact survey in the patient’s home, BCG will be given to all included contacts, followed by chemoprophylaxis with SDR two months later in those groups randomized to receive it (FU1). Follow-up examinations will be carried out one year (FU2) and two years (FU3) after receiving BCG. The three follow-up moments will be used to investigate whether the contact has developed leprosy or may be a suspected leprosy case (primary outcome measure). These patients will be sent to Nilphamari hospital or a local clinic for further investigation and treatment of leprosy. At these moments both groups will also be examined for adverse events following the BCG vaccination. Blood samples will be taken from 300 randomly chosen contacts for further molecular and immunological testing. Subjects not available for follow-up during the house visits will be contacted in order to plan another house visit. The trial started in July 2012 and will have duration of intake of 24 months. With a total observation period of 2 years after intake, the study will thus be completed after 48 months.

A separate database has been designed for the trial, which is linked to the database already in use at the RHP. Data are entered in the field onto purpose designed data sheets during clinic visits and contact group surveys. These data are sent to the RHP center in Nilphamari, where they are entered into the database. All paper forms are scanned and filed on hard disk and CD. The paper copies of the data will be retained for at least 15 years after completion of the study. An electronic copy of the database is sent to the department of Public Health of Erasmus MC in the Netherlands on a monthly basis. Modern back-up facilities are available at Nilphamari as well. Protection of privacy of patients in the database will be according to Erasmus MC standards.

### Blinding

Ideally, we would like to have set up a (double) blinded trial. However, this was not possible, since we have not been able to locate any company that could produce placebo tablets especially for this trial.

### Adverse effects

Rifampicin can give adverse events, such as gastro-intestinal complaints, skin rash, elevated liver enzymes, headache, dizziness, influenza-like syndrome, acute loss of kidney function, thrombocytopenia, asthma-like symptoms and shock [13]. Also, rifampicin can cause urine, saliva, tears and faeces to turn an orange or red colour. However, the chance of developing these symptoms is low, especially when giving a single dose of rifampicin only. In a previous trial, in which over 20,000 contacts of leprosy were given SDR, no adverse events were reported, apart from innocent red discoloration of the urine (for which the contacts were forewarned) [8,14].

Serious complications of BCG vaccination are uncommon. Although localized skin reactions occur frequently; less than one in 1000 people vaccinated develop significant local reactions, such as abscesses or regional lymphadenitis [14,15]. More serious adverse effects include osteitis, osteomyelitis and disseminated infection, but these are rare [16-18]. As many as 95% of BCG recipients have an insignificant, local reaction at the site of inoculation, however, lesions typically heal by three months with permanent residual scarring at the puncture site.

Both interventions (BCG and SDR), have separately been used widely in contacts of leprosy patients, with minimal adverse effects [8,19]. There is no reason to expect any serious difficulties from the combined interventions, as they will be given two months apart. However, strict monitoring of adverse events will take place in the trial. Leaflets containing information about the aims and the methodology of the trial, and describing potential adverse reactions will be given to all contacts included in the trial. These leaflets request that contacts report any suspected adverse reactions to the responsible researcher. The responsible researcher will then examine all contacts with reported adverse reactions. All contacts will also be examined two months, one year and two years after administration of the BCG vaccine. Data on adverse events is collected on the Contact Registration Forms of the trial. In the event of minor side effects, contacts will be referred to a State Tuberculosis Medical Officer for treatment, but the trial will not be stopped. In case of serious adverse effects the PI will stop the trial and initiate an individualized treatment scheme. All costs for treatment will be refunded.

### Data analyses

Statistical analyses will be done using SAS software. We use techniques for the analysis of survey samples to account for the clustering at the level of the index patient in the sample. Bivariate associations are investigated using “proc surveyfreq” and the Rao Scott χ2 instead of the Pearson χ2. We also use “proc surveylogistic” instead of the ordinary logistic regression procedure. We report odds ratios, but because of the low prevalence of the outcome these are comparable with relative risks. The number needed to treat (NNT) is calculated per subgroup of contacts. A significance level of 5% is used in all tests.

### Sample size calculation

In our power calculation, heterogeneity in the chance of contacts to develop clinical symptoms of leprosy was taken into account, but no major effect on the numbers needed was found. In the earlier COLEP trial [8] we found an incidence rate (IR) of leprosy among household contacts and direct neighbors of 4 per 1000 per year in the untreated group over the first two years. We hypothesize that in contacts receiving BCG only, this number will be the same in the first year or possibly increase slightly. Also based on the previous trial, we expect a 50% reduction through the SDR intervention (IR of 2 per 1000). On the basis of these figures (with α = 0.05 two-sided, power = 0.80), a total of about 10,000 contacts will be necessary in each group in order to detect reliably the expected protective effect of the BCG plus SDR combination of 50%, even taking into account an expected 10% loss to follow-up of contacts.

### Blood samples for analysis of host immune and gene profiles

Early detection of *M. leprae* infection (before clinical manifestations occur) is vital to reduction of transmission. However, current diagnosis relies on detection of clinical signs, since there are no tests available to detect asymptomatic *M. leprae* infection or predict progression to leprosy. Furthermore, although BCG vaccination and rifampicin chemoprophylaxis are both proven strategies for leprosy prevention, it is not known how the immune and genetic biomarker profiles are influenced by these (combined) interventions. Identification of such profiles will enable distinguishing pathogenic from protective biomarkers and lead to effective prophylactic interventions for leprosy.

In this study we intend to evaluate and optimize diagnostic tools for identification of individuals who should best be targeted for prophylactic treatment. In order to develop improved diagnostic tests based on reliable biomarkers that are detectable in blood samples, this study will analyze immune- and genetic host parameters in order to identify biomarkers that distinguish individuals controlling bacterial replication from those developing disease using the following assays:

1. Whole blood assays (WBA):Upon recruitment 4 ml venous blood will be drawn and used directly in three WBA, using tubes pre-coated with *M. leprae* WCS, ML2478/ ML0840 recombinant proteins or without stimulus. Each tube will be marked with a colored cap specific for one of these stimuli. After 24 hour incubation at 37°C, tubes will be frozen and stored for analysis of cellular markers [20] and/or analysis in field-friendly lateral flow assays for detection of Th1/Th2 cytokines as well as anti-PGL-I Ab [21].

2. Dual color Reverse Transcription Multiplex Ligation dependent Probe Amplification (dcRT-MLPA). From each individual venous blood (app. 2.5 ml) will be added to a PAXgene® tube and stored at −80°C. Total RNA will be extracted, purified and used to identify differential gene expression by dcRT-MLPA [22] using 179 selected target genes (Geluk A, Van Meijgaarden KE, Wilson L, Van der Ploeg- van Schip JJ, Bobosha K, Quinten E, Dijkman K, Franken KLMC, Haisma I, Haks MC *et al*: **Longitudinal Immune Responses and Gene expression Profiles during Development of Type 1 Leprosy Reaction**. in preparation).

Blood samples will be taken from 150 randomly selected contacts in both arms of the trial (total 300) 6 weeks after BCG vaccination (Figure 1). In addition, blood will be taken from any contact developing leprosy during the observation period of 24 months at the time of diagnosis before treatment. The aim of this part of the study is to identify:

1. Host immune responses and gene expression profiles specific for pathogenic as well as protective immune responses to *M. leprae* by comparison of profiles of patients *vs.* contacts.

2. Effect of chemo- and immunoprophylactic interventions on host immune responses and gene expression profiles and clinical disease by comparison of profiles of BCG-vaccinated *vs.* non-vaccinated contacts.

As part of our study on host immune and gene profiles in a non-intervention group, conducted by the IDEAL (Initiative for Diagnostic and Epidemiological Assays for Leprosy) consortium, similar blood samples will also be taken from a cohort of 500 new leprosy patients, 5,000 of their contacts, and from new cases of leprosy arising from this contact group during a 24-month observation period. As a referent group (endemic controls), 250 healthy individuals from the general population will be sampled as well.

### Preparations and process evaluation

The trial is conducted according to detailed research protocols that were developed in close consultation with the senior staff of RHP. In addition, an online Good Clinical Practice (GCP) course was completed by all Principal Investigators. All research assistants received training in research protocol procedures and giving BCG. They were also assisted in the field by the staff of the national EPI program when giving the BCG, until they were well enough trained to do this independently. Training (both theoretical and practical) was also given in the venapunction of blood for the additional immunological and transcriptional analyses to be performed later. All researchers have a professional background in the diagnosis and treatment of leprosy and received refresher courses on this.

Quality checks on all aspects of the data collection and entry are performed monthly and feedback on the results is given to the field staff and the data entry manager. For this purpose Erasmus MC has employed a medical doctor as independent Trial Monitor in Bangladesh to perform supervision tasks on a monthly basis to ensure optimal compliance to the study protocol.

## Discussion

Combined chemoprophylaxis and immunoprophylaxis is potentially a very powerful and innovative tool aimed at contacts of leprosy patients, which could reduce the transmission of *M. leprae* substantially. The trial intends to substantiate this potential preventive effect.

Childhood BCG vaccination and SDR both have a protective effect for leprosy in contacts of approximately 60% [7,8]. But if a contact who had previously received BCG vaccination also received SDR, the protective effect appears to be up to 80% [9]. However, the Brazilian trial [7] showed that there was an increased risk of tuberculoid leprosy in the first months after BCG vaccination, even though this was fully compensated later on. Because this trial was not conclusive, it is important to determine whether the excess cases in the first year after immunoprophylaxis can be prevented by chemoprophylaxis.

Evaluation of immune responses and gene expression profiles will allow identification of pathogenic versus (BCG-induced) protective host biomarkers and could lead to effective prophylactic interventions for leprosy using optimized tools for identification of individuals who are most at risk of developing disease.

The global number of new leprosy cases has remained constant over the past years, indicating that the transmission of leprosy in close contacts of new, untreated cases is still ongoing. The combined use of BCG and rifampicin will be a powerful to tool in routine leprosy control to interrupt the transmission of leprosy.

### Ethical approval

The national Research Ethics Committee (Bangladesh Medical Research Council) has approved the study protocol (Ref no. BMRC/NREC/2010-2013/1534).

## Competing interests

The authors declare that they have no competing interests. The BCG vaccine will be provided free of charge by the Government of Bangladesh.

## Authors’ contributions

All authors contributed to the design of the study and manuscript preparation. All authors have read and approved the final manuscript.

## Pre-publication history

The pre-publication history for this paper can be accessed here:

http://www.biomedcentral.com/1471-2334/13/456/prepub
